# Comparative Analysis of Prediction Models for Trawling Grounds of the Argentine Shortfin Squid *Illex argentinus* in the Southwest Atlantic High Seas Based on Vessel Position and Fishing Log Data

**DOI:** 10.3390/biology14010035

**Published:** 2025-01-04

**Authors:** Delong Xiang, Yuyan Sun, Hanji Zhu, Jianhua Wang, Sisi Huang, Shengmao Zhang, Famou Zhang, Heng Zhang

**Affiliations:** 1 Key Laboratory of Oceanic and Polar Fisheries, Ministry of Agriculture and Rural Affairs, East China Sea Fisheries Research Institute, Chinese Academy of Fishery Sciences, Shanghai 200090, China; xdl17852167218@163.com (D.X.);; 2 Laoshan Laboratory, Qingdao Marine Science and Technology Center, Qingdao 266104, China; 3 College of Marine Living Resource Sciences and Management, Shanghai Ocean University, Shanghai 201308, China; 4 College of Navigation and Ship Engineering, Dalian Ocean University, Dalian 116023, China

**Keywords:** Southwest Atlantic, *Illex argentinus*, AIS, deep learning, fishing ground prediction

## Abstract

To evaluate and compare the effectiveness of prediction models for Argentine squid *Illex argentinus* trawling grounds in the Southwest Atlantic high seas based on vessel position and fishing log data, this study used AIS datasets and fishing log datasets from fishing seasons spanning 2019–2024 (December to June each year). Using a spatial resolution of 0.1° × 0.1° and a monthly temporal resolution, we constructed two datasets—one based on vessel positions and the other on fishing logs. Fishing ground levels were defined according to the density of fishing locations, and combined with oceanographic data (sea surface temperature, 50 m water temperature, sea surface salinity, sea surface height, and mixed layer depth). A CNN-Attention deep learning model was applied to each dataset to develop *Illex argentinus* trawling ground prediction models. Model accuracy was then compared and potential causes for differences were analyzed. Results showed that the vessel position-based model had a higher accuracy (Accuracy = 0.813) and lower loss rate (Loss = 0.407) than the fishing log-based model (Accuracy = 0.727, Loss = 0.513). The vessel-based model achieved a prediction accuracy of 0.763 on the 2024 test set, while the fishing log-based model reached an accuracy of 0.712, slightly lower than the former, indicating the high accuracy and unique advantages of the vessel position-based model in predicting fishing grounds. Using CPUE from fishing logs as a reference, we found that the vessel position-based model performed well from January to April, whereas the CPUE-based model consistently maintained good accuracy across all months. The 2024 fishing season predictions indicated the formation of primary fishing grounds as early as January 2023, initially near the 46° S line of the Argentine Exclusive Economic Zone, with grounds shifting southeastward from March onward and reaching around 42° S by May and June. This study confirms the reliability of vessel position data in identifying fishing ground information and levels, with higher accuracy in some months compared to the fishing log-based model, thereby reducing the data lag associated with fishing logs, which are typically available a year later. Additionally, national-level fishing log data are often confidential, limiting the ability to fully consider fishing activities across the entire fishing ground region, a limitation effectively addressed by AIS vessel position data. While vessel data reflects daily catch volumes across vessels without distinguishing CPUE by species, log data provide a detailed daily CPUE breakdown by species (e.g., *Illex argentinus*). This distinction resulted in lower accuracy for vessel-based predictions in December 2023 and May–June 2024, suggesting the need to incorporate fishing log data for more precise assessments of fishing ground levels or resource abundance during those months. Given the near-real-time nature of vessel position data, fishing ground dynamics can be monitored in near real time. The successful development of vessel position-based prediction models aids enterprises in reducing fuel and time costs associated with indiscriminate squid searches, enhancing trawling efficiency. Additionally, such models support quota management in global fisheries by optimizing resource use, reducing fishing time, and consequently lowering carbon emissions and environmental impact, while promoting marine environmental protection in the Southwest Atlantic high seas.

## Simple Summary

This study explores the effectiveness of a CNN-Attention model using AIS vessel position data and fishing log data to predict fishing grounds for Argentine squid (*Illex argentinus*) in the Southwest Atlantic high seas. Traditional forecasts primarily rely on fishing log data, which suffer from delays and limited spatial resolution, failing to meet actual fishing needs. This research utilizes vessel position data with a spatial resolution of 0.1° × 0.1° and monthly temporal resolution, creating a more refined dataset. Compared to fishing log datasets that include marine environmental factors (like SST, SSH, and MLD), this new dataset offers higher timeliness and accuracy. The analysis revealed that, from January to April, both datasets yielded good predictions, but the model based on vessel position data performed poorly in December, May, and June compared to the fishing log model. This research not only provides a reliable forecasting method for Argentine squid fishing grounds but also offers insights for future marine habitat predictions and ecological protection. By integrating AIS vessel data and fishing logs, the study proposes an effective strategy to enhance forecast accuracy, supporting efforts against climate change and illegal fishing.

## 1. Introduction

The Southwest Atlantic is one of the world’s significant fishing regions, particularly for cephalopod and demersal fish resources [1]. The high primary productivity of the South American continental shelf and its slopes provides a favorable habitat for marine life in this region [2]. The convergence of the warm Brazil Current from the north and the cold Malvinas Current creates a complex and dynamic marine environment, making this region one of the most active oceanic areas worldwide [3,4]. Major commercially harvested species in this area include *Illex argentinus*, *Merluccius hubbsi*, and *Patagonotothen ramsayi*. Among these, *Illex argentinus*, commonly known as Argentine shortfin squid, is a primary target for distant-water fishing fleets from multiple countries [5,6]. This squid belongs to the order Teuthoidea, family Ommastrephidae, and genus *Illex* [7]. The fishing grounds for this squid are found on the continental shelf at depths of 30–100 m, while high-seas trawling grounds are typically located at depths of 100–200 m. Each year, the squid fishing grounds first emerge within Argentina’s Exclusive Economic Zone (EEZ) in shallow waters. As the sea surface temperatures in summer in the Southern Hemisphere rise above 11 °C from December onward, the squid migrate to deeper waters on the continental shelf to feed or spawn, spreading into the deeper high-seas waters beyond the shallow EEZ, establishing an international trawling or jigging fishery. The spawning activity of this species occurs in the convergence zone influenced by both currents [8], while feeding takes place near the colder Malvinas Current, meaning that this dynamic marine environment significantly impacts squid resources. With only one reproductive cycle in its lifespan, this squid dies after spawning. This biological trait makes the population primarily dependent on recruitment levels [9], which are further influenced by environmental factors like temperature, salinity, and ocean currents. Several studies have analyzed changes in squid resources and fishing grounds by combining oceanographic data, with sea surface temperature (SST) identified as a critical factor [6,10,11]. Sea surface height (SSH) and sea surface salinity (SSS) also influence resource levels and distribution. However, previous research on squid fishing grounds predominantly relied on fishing log data [3]. Since log data are recorded by fishing vessels during operations at sea, its acquisition is time-consuming, typically resulting in at least a year’s delay, and log data accuracy lacks sufficient verification and quality control. Errors or omissions due to human factors, such as misreporting by captains, can affect data quality and introduce bias when analyzing spatial–temporal fishing ground variations or constructing prediction models. With advancements in the satellite Automatic Identification System (AIS) and vessel satellite reporting technology [12], vessel position data offer a way to overcome these limitations, with data acquisition delays under 0.5 h, providing high timeliness. Satellite sensors can cover vast marine areas, automatically recording dynamic information, including vessel latitude, longitude, speed, and heading over extended periods [13]. With modern global positioning systems (GPSs), position monitoring is accurate to the meter level [14], greatly reducing the chances of human error, omission, misreporting, and data manipulation. Currently, vessel position data primarily originate from the AIS [15] and the Vessel Monitoring System (VMS) [16]. AIS is an automated system for vessel communication and tracking, allowing fishing vessels to transmit their position, speed, and heading to other vessels, coastal stations, and satellites in real time, usually via VHF radio for good nearshore coverage and through satellite AIS for remote areas. VMS, on the other hand, periodically transmits vessel position, speed, and heading to management centers, ensuring continuous tracking by fishery management authorities. In summary, AIS and VMS facilitate both fishery management and vessel safety monitoring, though AIS emphasizes real-time inter-vessel information sharing, while VMS focuses on regulatory oversight and long-term tracking. Vessel position data are widely applied in vessel identification [17], extraction of fishing status [18,19], and analysis of fishing effort intensity [20].

With advances in computing and artificial intelligence, deep learning models, such as Convolutional Neural Networks (CNNs) [21] and Support Vector Machines (SVMs) [22], have gained widespread application in fishing ground prediction. However, existing studies still predominantly use fishing log data, defining fishing ground levels using catch volume (Catch) or catch per unit effort (CPUE). Yang [23] and Sun [24] proposed using the density of fishing location data to define central fishing grounds, but did not incorporate deep learning techniques for prediction. In the high seas of the Southwest Atlantic, squid is harvested through both jigging and trawling. Given the region’s strong, complex currents, trawling vessels have higher efficiency and more active operation compared to jigging vessels [25]. However, the region’s oceanographic environment still considerably affects trawling grounds, highlighting the high economic value of real-time trawling ground prediction models based on deep learning and vessel position data.

As outlined, while oceanographic conditions strongly influence the distribution and location of the squid fishing grounds, previous studies have largely relied on fishing log data [3]. Even when fishing ground predictions have been validated for high accuracy, the delay in obtaining fishing log data and the fishing grounds’ short-term spatial–temporal variability (with movements of 10–60 nautical miles typically occurring within 3–7 days in response to environmental changes) often limits their practical applicability. Thus, integrating near-real-time vessel position data to assess fishing locations and resource levels at finer scales is needed. The objectives of this study are to (1) propose a new approach for identifying squid trawling grounds and resource levels in the Southwest Atlantic using vessel position data-based state detection and haul extraction algorithms; (2) analyze inter-factor influences and identify key environmental factors affecting fishing grounds through Spearman correlation analysis; and (3) construct deep learning-based prediction models for the squid fishery using both vessel position and fishing log datasets, comparing their predictive accuracy and applicability. This study aims to provide scientific guidance and practical insights for locating *Illex argentinus* trawling grounds.

## 2. Materials and Methods

### 2.1. Data Sources

#### 2.1.1. Vessel Position Data and Fishing Log Data

**Figure 1:**
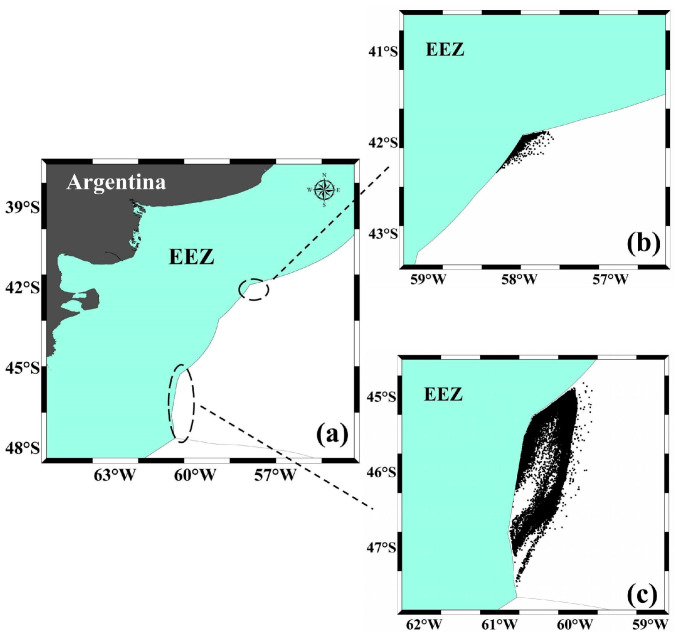
Location and distribution of *Illex argentinus* trawling grounds and fishing points in the Southwest Atlantic high seas, 2020–2024. (**a**) shows the location of the squid trawling grounds in the Southwest Atlantic high seas; (**b**) is a statistical plot of fishing points in the northern fishing ground; (**c**) is a statistical plot of fishing points in the southern fishing ground.

This study utilizes AIS vessel position data from 34 trawlers operating in the high seas of the Southwest Atlantic from 2019 to 2024. The vessel data used in this study all correspond to trawl fishing vessels operated by Chinese distant-water fishing companies in the Southwest Atlantic. All vessels employ bottom trawl gear, with lengths ranging from 45 to 65 m, and there is no significant difference in fishing efficiency. The vessel position data include details such as vessel name, timestamp, latitude and longitude, speed, and heading. Additionally, trawling speed and heading characteristics during trawling operations were recorded based on trawling speeds logged in fishing logs and real-time observations by trawler captains. Based on variations in speed and heading, three operational states were identified for the vessels: fishing, transit, and drifting. The main fishing grounds for the squid trawlers were located in the high seas between 40° W and 50° W and 55° S–60° S (Figure 1). The fishing season for this squid was defined as January to June each year, including December of the preceding year. For example, the 2021 fishing season spans December 2020 through June 2021, with monthly temporal resolution. Fishing ground levels were defined by calculating the density of trawling operations within each 0.1° × 0.1° grid cell from December to June each year. The median density was used to classify fishing ground levels: areas with values above the median were defined as central fishing grounds, while areas below the median were classified as general fishing grounds. The vessel position data were acquired through purchase from the authorized agent in Shanghai city in China of the USA AIS data service.

Fishing log data related to squid trawling operations in the Southwest Atlantic high seas were obtained from records kept by Chinese trawlers operating in the region during the fishing years between the 2020 and 2024. This dataset includes operational dates, set and haul locations, and catch quantities by species. The catch per unit effort (CPUE) for each 0.1° × 0.1° grid cell was calculated as the average catch per haul within that cell in fishing seasons over the five-year period. The median CPUE was used as the threshold to classify fishing ground levels, with values above the median indicating central fishing grounds and values below the median indicating general fishing grounds. The fishing logbooks, both in paper and electronic formats, were submitted by the relevant commercial fishing vessels to the East China Sea Fisheries Research Institute of the Chinese Academy of Fishery Sciences. After aggregation, these logbooks were provided to the researchers for subsequent data analysis.

#### 2.1.2. Oceanographic Data

Previous studies have shown that factors such as sea temperature, salinity, and ocean currents significantly impact the distribution of *Illex argentinus* fishing grounds [10,11], with the temperature at a 50 m depth identified as an important factor [6]. Therefore, the oceanographic data utilized in this study include sea surface temperature (SST), sea surface height (SSH), sea surface salinity (SSS), 50 m water temperature (50 m st), and mixed layer depth (MLD). Oceanographic data from 2020 to 2022 were sourced from the Asia-Pacific Data Research Center (http://apdrc.soest.hawaii.edu/las_ofes/v6/dataset?catitem=71 (accessed on 9 October 2024)), while data for 2023 and 2024 were downloaded from the Copernicus Marine Service (https://data.marine.copernicus.eu/products (accessed on 10 October 2024)). The spatial resolution of the Asia-Pacific Data Research Center data is 0.1° × 0.1° with a monthly temporal resolution, whereas the Copernicus Marine Service data have a spatial resolution of 0.083° × 0.083° with a monthly temporal resolution.

The study area was defined as the region between 35° W and 55° W and 50° S–70° S. The environmental data for this region were processed using Matlab R2023b to achieve a monthly temporal resolution and a 0.1° × 0.1° spatial resolution.

### 2.2. Data Processing Methods

#### 2.2.1. Fishing Activity Classification

The threshold method was used to classify vessel activities during the fishing season. This traditional approach leverages trajectory information to identify vessel status and has been widely used for fishing vessel classification [26,27]. Although the fishing logbooks record information for each fishing haul, the vessel speed data are limited to the speed at the time of recording. Since the vessel speed is not constant during trawling operations, relying solely on the speed information from the logbook for threshold state determination presents significant limitations. Information about trawling activities in the Southwest Atlantic high seas was obtained through interviews with captains, specifically focusing on trawling posture and behavior. Using AIS data for vessel position, heading, speed, and actual trawling speed records from fishing logs (3.6–5.5 knots), we applied a manual classification based on speed and heading variations. Based on insights from fishery observers in the Southwest Atlantic, vessel behavior was categorized into three states:

State 1: Fishing—characterized by a speed between 3.6 and 5.5 knots, with a speed difference of less than 1.5 knots between two consecutive AIS reports, and a heading difference of less than 30°.

State 2: Sailing—defined by a speed above 5.6 knots, or a speed between 3.6 and 5.5 knots with a speed difference greater than 1.5 knots or a heading difference exceeding 30° between consecutive positions.

State 3: Drifting—identified by a speed below 3.5 knots, where the heading is influenced by the combined direction of currents and wind.

**Figure 2:**
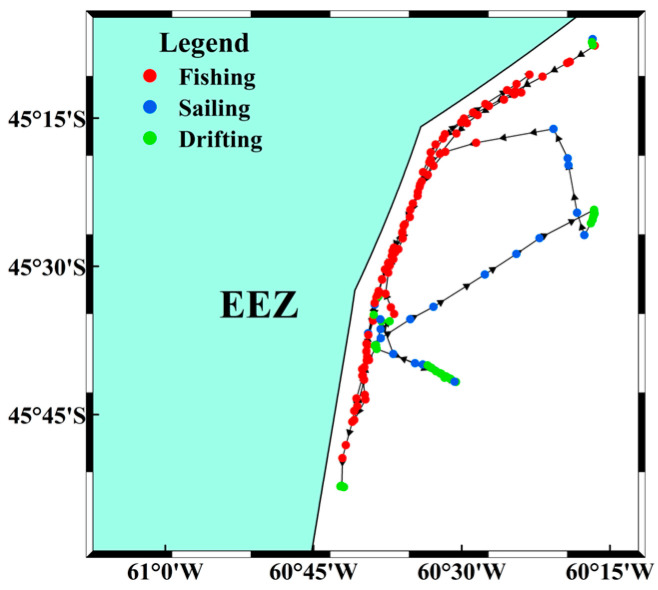
Illustration of trawler status classification using the threshold method (example of vessel Luqingyuanyu 201 with MMSI 412,329,684 from 00:00 on 1 January to 24:00 on 2 January 2023).

After classifying vessel activities (Figure 2), the dataset was filtered. When consecutive fishing states exceeded two hours, it was defined as a single trawling operation. The midpoint position (or the nearest position to this time) was taken as the central latitude and longitude for that trawl, and monthly trawl frequencies were calculated within each 0.1° × 0.1° grid cell. Over four fishing seasons, vessel position data yielded a total of 39,264 trawling operations, of which 38,756 were recorded in fishing logs, indicating high accuracy in trawl extraction based on vessel positions. After completing vessel activity classification, the monthly total fishing duration was calculated for each year. For each month, the average daily fishing duration was determined using fishing log data and AIS data for the three states. The average daily fishing duration in the logs was derived from the time difference between set and haul operations, while the total time spent in each state was calculated for each occurrence. The average daily duration was then obtained by calculating the mean.

For calculating the CPUE from fishing logs, a spatial resolution of 0.1° × 0.1° was applied. The average fishing effort for all trawling operations within each fishing area was calculated monthly, as shown in Equation (1):

(1) $${CPUE}_{i,j,t}=\frac{\sum_{k=1}^{n_{i,j,t}}C_{i,j,t,k}}{\sum_{k=1}^{n_{i,j,t}}E_{i,j,t,k}}$$

In the formula, i, j represents the sequence of each fishing area defined by latitude and longitude, t denotes a specific time period, C_i,j,t,k_ is the catch amount (in tons) for the kth fishing operation during a specific ten-day period in that fishing area, and E_i,j,t,k_ corresponds to the fishing effort for the same operation. Additionally, n_i,j,t_ indicates the number of fishing operations in that area during the specified ten-day period.

After calculating the trawl operations’ frequency from vessel position data and CPUE from fishing logs for each area, fishing grounds were classified based on their distributions. Using the median as a threshold, areas with values above the median were designated as central fishing grounds, while those below the median were classified as general fishing grounds.

#### 2.2.2. Correlation Analysis

After classifying fishing grounds in each area, the corresponding oceanographic data for each time period were extracted and matched with the latitude and longitude of each fishing ground to construct a dataset. This dataset includes nine dimensions: year, month, longitude, latitude, SST, 50 m st, SSH, SSS, and MLD. To evaluate the strength and direction of relationships among these variables, assess their interdependencies, and analyze recent trends in the oceanographic environment of the Southwest Atlantic, a correlation analysis was conducted on the dataset. Commonly used correlation analysis methods include Kendall [28], Pearson [29], and Spearman [30]. Given that the dataset, derived from vessel-based fishing ground centroids and oceanographic data, does not follow a normal distribution and consists of point data without inherent order consistency, Spearman’s rank correlation coefficient was chosen for the correlation analysis. This method was applied to assess the relationships between the variables, as it is well suited for handling nonlinear relationships and non-normal distributions.

#### 2.2.3. CNN-Attention Model

**Figure 3:**
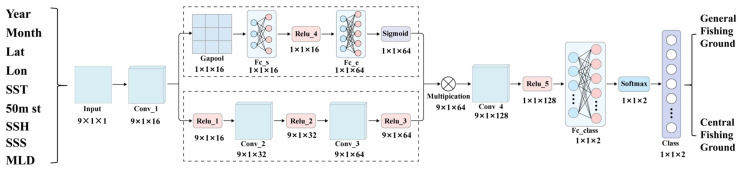
Structure of the CNN-Attention model used in this study (revised from [33]).

This study developed a deep learning prediction model for *Illex argentinus* fishing grounds using Convolutional Neural Networks (CNNs) [31] combined with an Attention mechanism [32], implemented in the Matlab programming language. The model aims to accurately predict the regions and occurrence times of central and general fishing grounds, providing valuable guidance for fishery management. The CNN-Attention model was constructed using nine environmental indicators from the dataset as input factors, aiming to distinguish between central and general fishing grounds and analyze their relationships with the input factors. Data from the 2020 to 2023 fishing years were used as the training and validation sets (split 8:2), while data from 2024 were used as the test set to evaluate the model’s prediction performance. The model framework (Figure 3) is based on that described in the literature [33].

The model’s input data consist of spatiotemporal information across nine factors, with a 9 × 1 × 1 dimension corresponding to different spatial or temporal environmental conditions. First, the input data undergo an initial convolutional layer for feature extraction, generating a 9 × 1 × 16 feature map to capture local patterns across spatial dimensions. The model then processes the feature map through two parallel paths. In the first path, global average pooling (GAP) reduces the feature map to a 1 × 1 × 16 dimension, followed by two fully connected layers (Fc_s and Fc_e) with output dimensions of 1 × 1 × 16 and 1 × 1 × 64, respectively, representing global environmental characteristics influenced by multiple factors. ReLU and sigmoid activation functions are used in this path to extract nonlinear features and capture critical attention information, enabling the model to dynamically weigh specific environmental factors. In the second path, two convolutional layers (Conv_2 and Conv_3) output feature maps with dimensions of 9 × 1 × 32 and 9 × 1 × 64, respectively, to capture deeper interactions between factors. Two ReLU activations further enhance the model’s understanding of complex, nonlinear combinations of multidimensional environmental factors. The outputs from both paths are then element-wise multiplied to create a 9 × 1 × 64 fused feature map. This fusion step, facilitated by the Attention mechanism, focuses the model’s attention on key environmental factors that are more relevant to fishing ground classification. The fused feature map then undergoes additional feature extraction through another convolutional layer (Conv_4), resulting in a 9 × 1 × 128 feature map. A fully connected layer (Fc_class) compresses this to a 1 × 1 × 2 output, which then undergoes Softmax activation to produce the final classification result between central and general fishing grounds.

The CNN-Attention models, constructed separately using AIS vessel position data and fishing log data, were employed to analyze fishing grounds. Comparative analyses of accuracy and loss rate trends were conducted to evaluate each model’s predictive accuracy and generalization capability. Using both datasets from 2024 as test sets, fishing ground forecasts for 2024 were generated, with predictive performance metrics—including accuracy, precision, recall, and F1-score—used to assess and compare model outcomes [21]. Given the differing criteria for fishing ground classification—where the vessel position data model classifies fishing ground levels based on trawl density, and the fishing log model relies on CPUE values—further validation of model performance in practical fishing scenarios was conducted. CPUE, calculated from actual catch records in fishing logs, served as the standard for assessing actual fishing ground levels. At a monthly resolution, the accuracy of each model’s predicted fishing ground classifications was compared with actual fishing ground levels.

## 3. Results

### 3.1. Fishing State Classification and Fishing Ground Level Determination

**Figure 4:**
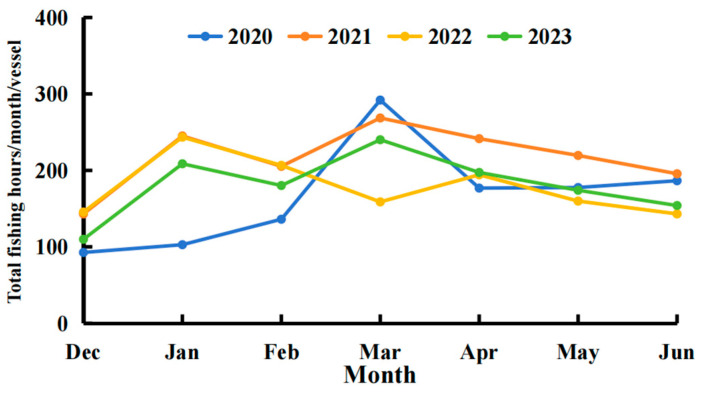
Monthly total fishing duration per vessel during the 2020–2023 fishing seasons based on AIS data.

After applying the threshold method to classify vessel positions during the squid fishing seasons from 2020 to 2023, the monthly trawling duration was calculated (Figure 4). There was considerable fluctuation in the monthly trawling duration trends each year. In the 2020 fishing season, trawling duration increased from December of the previous year to March of the current year, peaking in March. The total monthly trawling time per vessel in March was 3.15 times that of December. After reaching this peak, the trawling duration dropped sharply in April, with values from April to June ranging between 0.61 and 0.63 times that of March.

The trends for 2021–2023 showed two peaks each year. The patterns in 2021 and 2023 were similar: trawling duration increased from December to January, decreased in February, reached a second peak in March, and then steadily declined until June. The monthly trawling duration in 2021 was higher than in 2023, with the smallest difference in February (25.1 h) and the largest difference in May (45.3 h). In 2022, the pattern differed from 2021 and 2023, with the second peak occurring in April, resulting in two peaks in January and April. The total trawling duration in these two months was 1.68 and 1.33 times that of December, respectively, with trends similar to those observed in 2021 and 2023.

**Figure 5:**
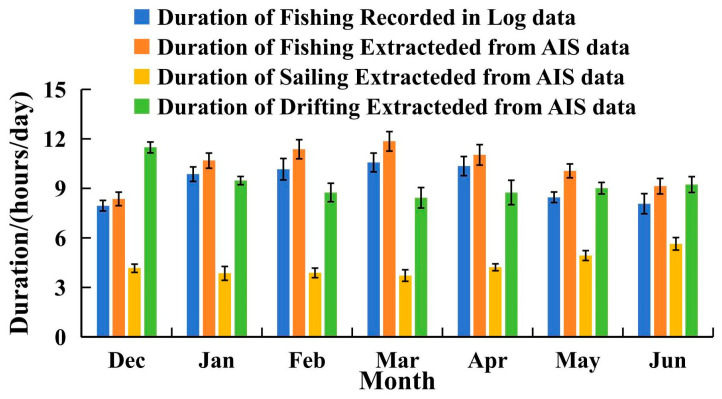
Monthly average duration (hours/day/vessel) on fishing (from AIS and Logs), sailing, and drifting behavior for trawlers during the 2020–2023 fishing seasons.

Using set and haul times recorded in the fishing logs, the average daily fishing duration for each month over the four fishing seasons was calculated and compared with the monthly average daily duration classified into the three AIS-based states (Figure 5). It was observed that the average daily fishing duration extracted from the logs was slightly lower than that determined from AIS data, though both showed similar trends. From December (average daily fishing duration of 7.95 h from logs, compared to 8.35 h from AIS) to March (10.67 h from logs, 11.85 h from AIS), the fishing duration showed an increasing trend, which then declined steadily until June (8.07 h from logs, 9.13 h from AIS). The sailing duration based on AIS data displayed minor fluctuations from December to April (ranging between 3.72 and 4.22 h) but increased significantly in May and June, reaching 4.93 and 5.64 h, respectively. Drifting duration was longest in December (11.48 h), with a declining trend from December to March (8.43 h), followed by an increase from March through June. This trend in drifting duration was inversely correlated with fishing duration.

**Figure 6:**
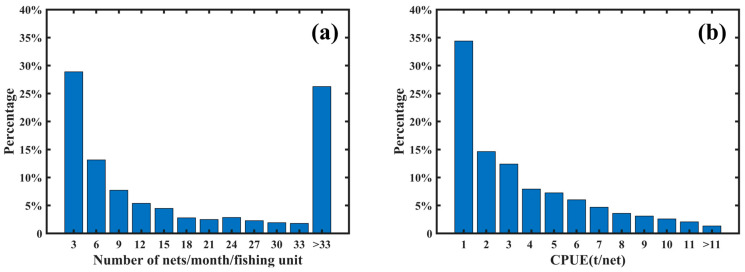
Proportional distribution of monthly trawling occurrences and CPUE values within grid cells in these squid fishing grounds from 2020 to 2023 ((**a**): proportion of trawling occurrences (%), (**b**): proportion of CPUE values (%)).

Fishing ground datasets were constructed separately based on vessel position and fishing log data, with a temporal resolution of one month and a spatial resolution of 0.1° × 0.1°. In the vessel position dataset, each trawl operation was represented by the central location of the fishing position at the midpoint time of the operation. Fishing ground levels were defined by counting the number of times each area recorded a central fishing position within a month. For the fishing log dataset, fishing ground levels were defined by calculating the CPUE for each area per month using Equation (1) (Figure 6). In the vessel position dataset, areas with a single fishing center occurrence were the most common, comprising about 29% of the dataset. As the number of fishing occurrences increased, the number of areas decreased. Based on the distribution, an area with three occurrences per month was used as the median. Areas with nine or fewer occurrences in a month were classified as general fishing grounds, while areas with more than nine occurrences were defined as central fishing grounds. In the fishing log dataset, areas with a CPUE of less than 1 ton per trawl were the most common, accounting for about 35% of the dataset. As CPUE values increased, the number of areas decreased. Based on the distribution, a CPUE value of 2 tons per trawl was used as the median. Areas with a monthly CPUE of 2 tons per trawl or less were classified as general fishing grounds, while areas with a CPUE above 2 tons per trawl were defined as central fishing grounds. Areas with a monthly CPUE of 2 tons per trawl or less were classified as general fishing grounds, while areas with a CPUE above 2 tons per trawl were defined as central fishing grounds.

### 3.2. The Relationship Between Time, Space, and Environmental Factors

**Figure 7:**
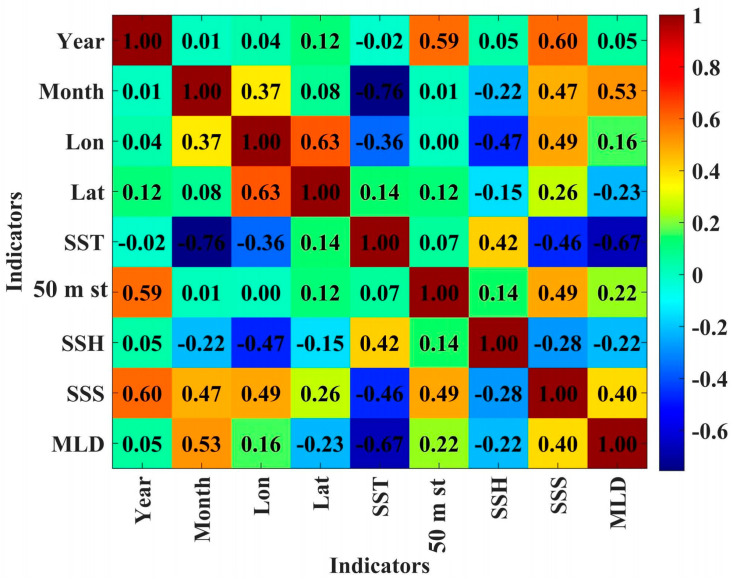
Heatmap of Spearman correlation analysis for nine indicators.

Using the Spearman function, a correlation analysis was performed on nine indicators, with a heatmap visualizing the resulting correlation matrix (Figure 7). The correlation coefficients range from [−1, 1], rounded to two decimal places, where 0 indicates no correlation between two indicators, positive values indicate a positive correlation, and negative values indicate a negative correlation. Higher absolute values denote stronger correlations.

In the correlation analysis, SST displayed significant variations in correlation with other variables, particularly with Month. The correlation coefficient between SST and Month was −0.76, indicating a strong negative correlation, suggesting that SST exhibits significant seasonal fluctuations over time. Additionally, SST and SSH showed a moderate positive correlation with a coefficient of 0.42, implying that temperature fluctuations may be somewhat related to sea surface height variations. SST and SSS had a correlation coefficient of −0.46, suggesting that as SST rises, SSS tends to decrease, showing a moderate negative correlation. The correlation between SST and MLD was also notable, with a coefficient of −0.67, indicating a trend toward a shallower MLD as SST increases. The 50 m st value was moderately positively correlated with Year, with a coefficient of 0.59, indicating a trend of rising temperatures at this depth over time. Correlations between 50 m st and other variables, such as Month, SSH, SST, SSS, and MLD, were relatively weak, with correlation coefficients around 0.1 and not exceeding the 0.4 threshold, suggesting limited significance. This result implies that temperature changes at 50 m are more influenced by temporal factors rather than other oceanographic parameters. SSS showed a significant negative correlation with Year, with a correlation coefficient of −0.60, indicating a declining trend in SSS over time. SSS also showed a moderate positive correlation with Lon, with a coefficient of 0.49, indicating regional differences in salinity related to geographical position. Additionally, SSS had a moderate positive correlation with Month (0.47), suggesting that seasonal changes may influence salinity throughout the year. The negative correlation between SSS and SST (−0.46) implies that salinity may decrease as temperature rises. SSH showed relatively weak correlations with other variables, but it did exhibit a moderate positive correlation with SST, with a coefficient of 0.42, suggesting that there may be synchronous fluctuations between temperature and sea surface height. Correlations between SSH and Year, Month, 50 m st, SSS, MLD, and other variables did not exceed 0.4, indicating a lack of significant direct linear relationships between these variables and sea surface height. MLD showed a moderate positive correlation with Month, with a coefficient of 0.53, indicating seasonal variations in MLD across different months. Additionally, MLD had a significant negative correlation with SST (−0.67), suggesting that rising surface temperatures may coincide with a reduction in MLD. MLD correlations with other variables, such as Year, Lon, Lat, 50 m st, SSH, and SSS, were below 0.4, suggesting these factors have limited direct impact on MLD.

Overall, the Spearman analysis revealed distinct correlations between SST, 50 m st, SSS, SSH, and MLD with other factors, with notable relationships between SST and Month, SST and MLD, and a positive correlation between 50 m st and Year. Additionally, SSS correlations with Year, Lon, and Month suggest that geographical and temporal factors influence salinity changes. The moderate positive correlation between SSH and SST indicates that temperature changes may affect sea surface height, while MLD variations are primarily influenced by SST and Month. These findings reveal the complex interrelationships among various physical parameters in the marine environment.

### 3.3. Accuracy and Prediction Validation of the CNN-Attention Model

**Figure 8:**
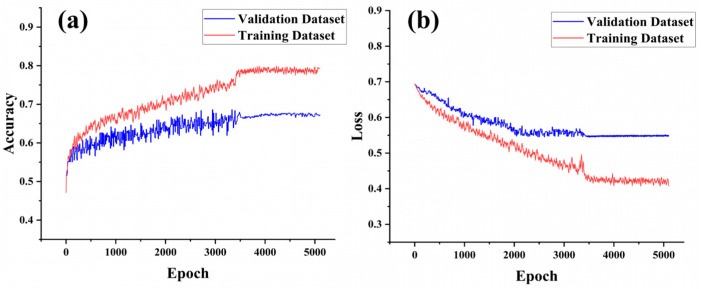
Accuracy and loss rates for the training and validation sets using vessel position data in the CNN-Attention model. ((**a**) shows the accuracy trends of the training and validation datasets, (**b**) shows the loss trends of the training and validation datasets).

**Figure 9:**
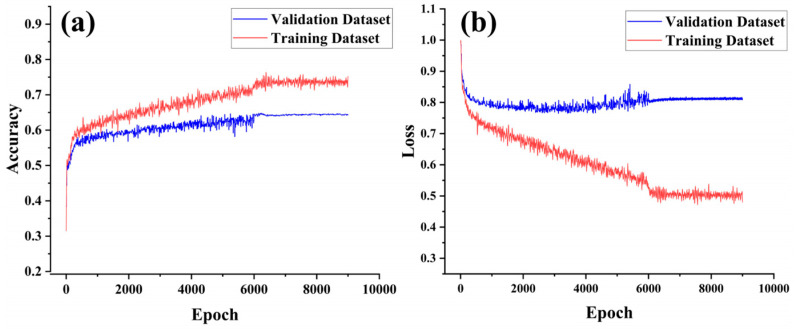
Accuracy and loss rates for the training and validation sets using fishing log data in the CNN-Attention model. ((**a**) shows the accuracy trends of the training and validation datasets, (**b**) shows the loss trends of the training and validation datasets).

The CNN-Attention model can automatically extract and learn both local and global features from input data. Particularly when data exhibit spatial or temporal correlations, the convolutional layers of CNN can capture more complex feature interactions, and the Attention mechanism enhances the model’s dynamic weighting capability. Thus, the CNN-Attention model has a significant advantage in capturing complex patterns and multidimensional associations. Vessel position data and fishing log data were used to construct separate datasets, which were input into the model for training. The accuracy and loss trends during training were recorded (Figure 8 and Figure 9) to compare the quality of the two datasets.

When comparing the accuracy and loss trends of the CNN-Attention model trained on vessel position data versus fishing log data, it was observed that the model constructed with vessel position data (Figure 8) slightly outperformed the model built with fishing log data (Figure 9). For the vessel position data model, both training and validation set accuracy increased rapidly in the early training phase (first 500 epochs), demonstrating strong learning capability. The model converged around 3400 epochs, and after 5100 epochs, the validation accuracy stabilized at approximately 0.674, while the training accuracy reached close to 0.813. At this point, the validation loss decreased to 0.542, and the training loss to 0.407. For the fishing log data model, accuracy for both the training and validation sets increased rapidly in the early training phase (first 500 epochs). The model converged around 6000 epochs, and after 9000 epochs, the validation accuracy stabilized at approximately 0.624, while the training accuracy reached close to 0.727. At this stage, the validation loss decreased to 0.822, and the training loss to 0.513.

The high accuracy on the training set for both models indicates strong fitting capability to the training data. Comparing Figure 8a and Figure 9a, the vessel position data model’s validation accuracy remained stable with minimal fluctuations, suggesting strong generalization across different data samples. In contrast, the fishing log data model showed more fluctuation in validation accuracy, especially after 2000 epochs, indicating a potentially weaker adaptation to this dataset. Figure 8b and Figure 9b show the changes in loss values for the training and validation sets over training epochs for both datasets. For the vessel position data model, loss values decreased rapidly and stabilized after 3400 epochs, with the final training loss around 0.4 and the validation loss around 0.54. In comparison, the fishing log data model’s loss values decreased more slowly during early training, converging only after 6000 epochs. Although the trend eventually stabilized, the final validation loss remained around 0.82, indicating possible underfitting on this dataset. Furthermore, the vessel position data model exhibited a smaller gap between training and validation loss values, whereas the fishing log data model showed a more pronounced gap, further suggesting that the former had stronger generalization capability.

**Figure 10:**
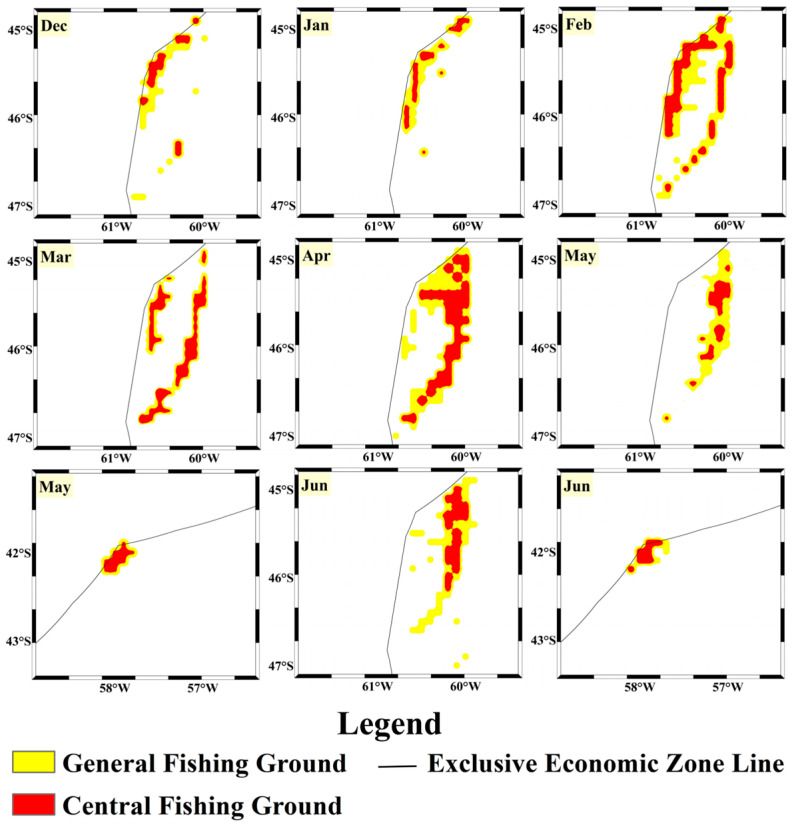
Predicted distribution of the squid high-seas trawling grounds based on the AIS vessel position data model.

**Figure 11:**
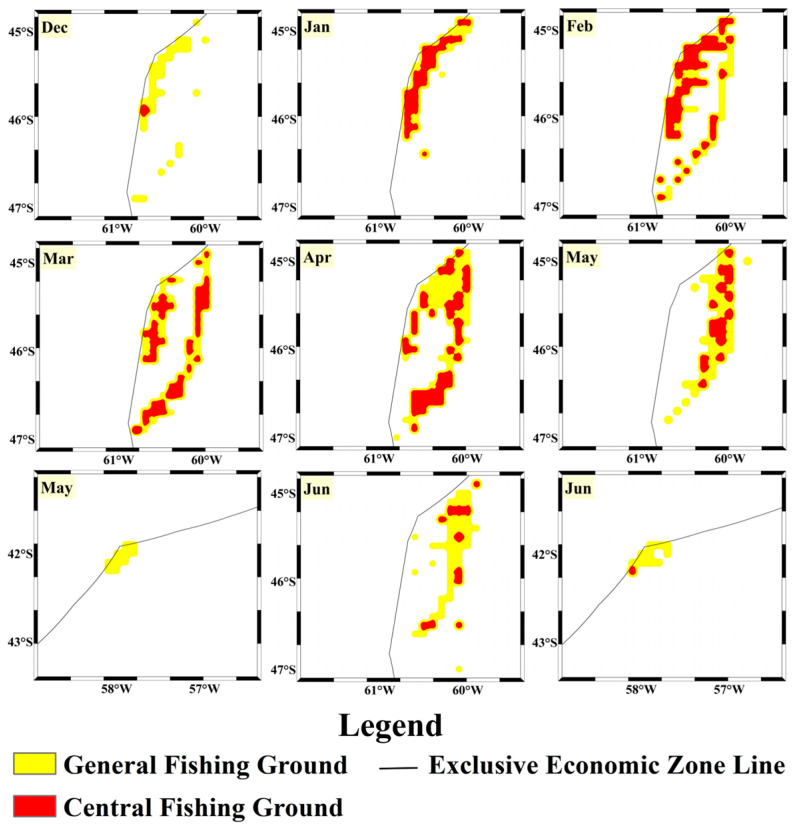
Predicted distribution of the squid high-seas trawling grounds based on the fishing log data model.

Based on the accuracy and loss rates of both datasets in the model, it can be concluded that the CNN-Attention model trained with vessel position data outperforms the model trained with fishing log data in the fishing ground classification task. This model demonstrates not only a high fitting capacity on the training set but also strong performance on the validation set, allowing for better prediction of fishing ground levels. To further validate the model’s predictive capabilities, the 2024 data were used as the test set. Predictions of the squid fishing ground levels and locations were made on a monthly scale, visualized by month (Figure 10 and Figure 11), and evaluated using accuracy, precision, recall, and F1-score as performance indicators.

Using the density of trawling operations as the criterion for fishing ground classification, the vessel position data model predicted the spatial and temporal variation in fishing grounds in 2024 (Figure 10). Results indicate that in December 2023, no significant central fishing grounds appeared, suggesting that the species might have been primarily distributed within the Exclusive Economic Zone (EEZ). Starting in January, a substantial range of central fishing grounds emerged and continued to expand, peaking in March, where the central fishing ground area was 2.73 times that of January. February and April also had relatively large central fishing ground areas, at 2.4 and 2.17 times the size of January, respectively. In May, the central fishing ground area significantly decreased to 1.43 and 1.6 times that of January and April, respectively. The *Illex argentinus* population in the high seas of the Southwest Atlantic first appeared near the EEZ boundary at 45° S–46° S, showing a slight northward movement from January to February. After February, the population gradually moved southeast to deeper waters, reaching near the 42° S EEZ boundary in May and June. For the test set, the vessel position data model achieved an accuracy of 0.759, precision of 0.781, recall of 0.751, and F1-score of 0.765, indicating that the CNN-Attention model constructed with vessel position data has good predictive accuracy for trawling density.

For the fishing log data model, CPUE was used as the criterion for fishing ground classification to predict the spatial and temporal variation in fishing grounds in 2024 (Figure 11). Its predictions were mostly similar to those of the vessel position data model, with both models predicting similar large-scale central fishing ground location patterns. However, the fishing log data model predicted smaller central fishing ground areas in December and June, indicating that while trawling activity was higher at the start and end of the fishing season, the actual catch yield was lower. For the test set, the fishing log data model achieved an accuracy of 0.664, precision of 0.657, recall of 0.735, and F1-score of 0.694. Although the CNN-Attention model built with fishing log data also shows good predictive performance, its evaluation metrics are lower than those of the vessel position data model. This discrepancy may be due to the vessel position data model’s difficulty in distinguishing the abundance of a single species, which may have led to overestimation of squid fishing ground levels during certain months.

**Figure 12:**
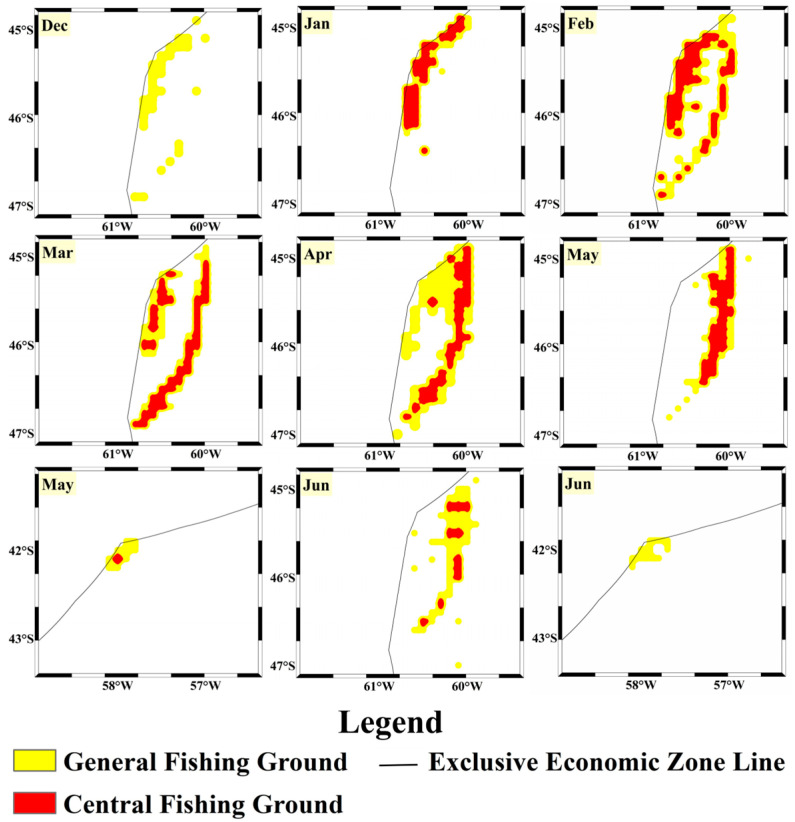
Real Distribution of the squid high-seas trawling grounds based on the 2024 year CPUE determination.

**Figure 13:**
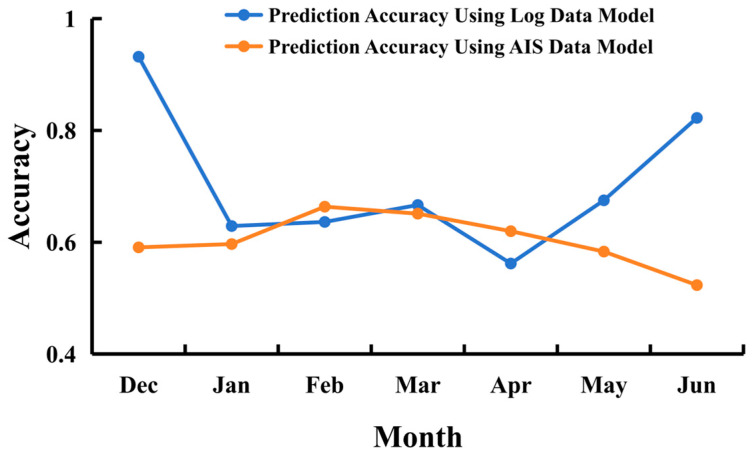
Monthly prediction accuracy of the vessel position data model and fishing log model.

To further compare the predictive performance of the two models with actual fishing operations, we used the CPUE calculated from the 2024 fishing log records as the standard for determining actual fishing ground levels, visualized in Figure 12. The predictive performance of each model was compared to actual fishing ground levels, and the monthly accuracy of both models was calculated based on the agreement between predicted and actual fishing ground levels (Figure 13). According to actual fishing ground levels based on CPUE, there were no central fishing grounds in the high seas area in December 2023, and the area of central fishing grounds was also small in June 2024. The log data model predicted only a small central fishing ground area in December and a similarly reduced area in May, aligning well with actual data. For the rest of the year, the actual central fishing ground scale and variation patterns closely matched the predictions from both models. The fishing log model showed higher accuracy in December and May–June when compared to actual fishing grounds, and it performed well in other months as well. The vessel position model, however, had lower accuracy in December and May–June, likely because the fishing grounds during these months were less productive, with many trawling operations yielding minimal catches. Therefore, trawling density was not a reliable indicator of fishing ground quality during these months. For other months, the vessel position model’s predictions were more accurate, indicating that during the peak fishing season from January to April, when the squid fishing grounds are concentrated and resource-rich, trawling density is positively correlated with CPUE.

## 4. Discussion

### 4.1. Fishing State Classification Based on Vessel Position Data

For extracting fishing states from vessel position data, the temporal range and spatial locations largely matched the information recorded in fishing logs, indicating that the threshold values determined through interviews with fishermen were reasonable. However, there were some discrepancies; the fishing log data may have subjective inaccuracies in spatial and temporal records due to manual entry, while vessel position data also have spatial position inaccuracies [34]. The threshold method has been widely applied in previous studies to classify fishing vessel states [35,36]. By setting appropriate thresholds, this method effectively distinguishes fishing from non-fishing activities, enhancing the accuracy of vessel state classification. Additionally, the threshold method simplifies the data analysis process, enabling efficient processing and classification of large volumes of vessel position data.

An analysis of fishing duration based on fishing states identified by the threshold method showed that fishing duration in 2021 and 2023 followed similar trends, while large-scale fishing activities in 2020 did not commence until March. In 2022, January and April were the months with the longest fishing duration. These trends align with the findings of Xiang [6] on CPUE fluctuations in Southwest Atlantic trawling vessels, where CPUE was low from December 2019 to January 2020, and then increased after March. Similarly, CPUE dropped after January 2022, only to rise again by late March. This indicates that fishing effort identified from vessel position data largely reflects actual fishing intensity [23], and changes in fishing intensity also show a degree of overlap with CPUE trends.

A comparison of monthly average daily duration for each trawler state revealed that fishing duration calculated based on logbook start and end times was consistently lower than the fishing duration derived from vessel position data. This discrepancy could be due to fishermen recording information approximately half an hour after each trawl ends, rather than immediately [34]. The duration of each state derived from vessel position data also reflects changes in resource abundance throughout the fishing season. In December, fishing grounds had not yet formed at a significant scale, so fishing time was relatively short and drifting time was longer. From January to April, squid resources were more abundant, forming stable central fishing grounds, leading to longer daily fishing times, with March recording the highest average daily fishing time. After March, fishing time gradually decreased, while drifting time increased, reflecting peak squid resource abundance in March. In May and June, sailing time increased significantly compared to previous months, indicating a more dispersed squid distribution. A northern fishing ground formed near the 42° S EEZ boundary, requiring vessels to increase sailing time to travel between northern and southern fishing grounds, as well as across multiple areas within the same fishing ground.

### 4.2. Ocean Environment Variability and Its Impact on Fishing Grounds

Various ocean environmental variables often exhibit non-normal distributions and outliers. By using the Spearman function for correlation analysis among indicators, we can effectively avoid bias due to nonlinear relationships or outliers among the indicators, revealing the complex relationships among variables such as SST, SSH, and MLD.

In analyzing SST’s correlation with other indicators, pronounced seasonal fluctuations are particularly evident. The strong negative correlation between SST and Month (−0.76) indicates significant seasonal variation in sea surface temperature over time. This relationship likely corresponds to the cyclical rise and fall of temperatures across seasons, with SST peaking in summer and reaching lower levels in winter. Additionally, the moderate positive correlation between SST and SSH (0.42) may reflect a trend of rising sea surface height as temperatures increase, which could be partially attributed to thermal expansion effects [37]. When water temperature rises, ocean water volume expands, leading to an increase in sea surface height. This effect is particularly noticeable over large marine areas where temperature increases can cause vertical expansion of water columns. The significant negative correlation between SST and MLD (−0.67) further reveals the influence of surface temperature on water column structure [38]. As SST rises, the surface water warms, reducing its density and resulting in a shallower mixed layer depth. This effect is especially prominent in summer when higher temperatures stabilize the water column, inhibiting vertical mixing and decreasing MLD. The significance of this relationship suggests SST’s key role in regulating ocean vertical structure, with important implications for the distribution of marine life and nutrients. The positive correlation between 50 m st and Year (0.59) indicates a temporal warming trend at this depth, reflecting the long-term impact of global warming [39]. With the warming trend induced by climate change, water temperature at 50 m gradually increases, possibly due to intensified heat exchange between the atmosphere and surface waters, which gradually impacts deeper layers. This trend has been observed in multiple regions globally, indicating that the rise in 50 m st aligns with the long-term increase in atmospheric temperatures. However, the weak correlation of 50 m st with other factors suggests that its changes are primarily driven by temporal factors, with limited direct influence from other oceanographic parameters. The negative correlation between SSS and Year (−0.60) implies a decreasing trend in sea surface salinity over time, potentially related to climate-induced changes in the evaporation–precipitation balance [40]. In some regions, as the climate warms, increased precipitation may lead to a decrease in salinity, especially in tropical and subtropical zones. Additionally, the positive correlation of SSS with Longitude (0.49) and Month (0.47) indicates that salinity distribution is influenced not only by temporal factors but also by geographical location and seasonal changes. Longitude-related variations may reflect the impact of different regional ocean circulation patterns, while the influence of Month suggests the regulatory role of seasonal factors, such as monsoons, precipitation, and evaporation, on salinity. The positive correlation between SSH and SST (0.42) may be associated with the thermal expansion caused by rising sea surface temperatures [37]. When SST increases, rising water temperatures reduce density, causing the water volume to expand and increasing the sea surface height. This effect is particularly noticeable over large ocean areas, especially in tropical regions where SSH fluctuates more significantly with temperature variations. However, the weak correlation between SSH and other factors indicates that changes in sea surface height are mainly controlled by localized ocean temperature and thermal expansion effects, with limited direct influence from other environmental factors. The positive correlation between MLD and Month (0.53) shows that mixed layer depth varies seasonally, likely reflecting thermodynamic changes in the ocean surface layer across seasons [41]. For instance, in summer, higher temperatures cause a shallower MLD, as increased temperatures stabilize the water column, inhibiting vertical mixing. In winter, surface temperatures drop, enhancing cooling processes that promote vertical mixing, thereby deepening the MLD. The negative correlation between MLD and SST (−0.67) further supports this phenomenon, indicating that higher surface temperatures are associated with shallower mixed layers. Overall, the seasonal variation in MLD is not only related to SST but also to seasonal fluctuations in atmospheric temperatures and ocean thermal processes.

The correlation relationships among ocean environmental factors reflect the multiple interactions within the marine environment. SST, as a key influencing factor, has both direct and indirect effects on SSH and MLD, and its strong correlation with Month highlights significant seasonal variations. The variability of 50 m st is primarily driven by long-term climate change trends, while SSS is influenced by temporal, geographical, and seasonal changes. These results provide important scientific insights into the relationships between physical parameters in the ocean, contributing to a deeper understanding of marine system responses in the context of climate change. Changes in *Illex argentinus* fishing grounds are mainly influenced by variations in ocean environmental factors. This study found that the optimal SST range for fishing grounds is between 9.7 °C and 14.8 °C, typically occurring from January to April each year. However, as SST drops below 9 °C after May, central fishing grounds may still appear, possibly because the squid’s tolerance to lower temperatures increases as they mature. Additionally, after May, fishing grounds are mostly located in deeper waters, suggesting that deeper water temperatures may have a greater impact on fishing grounds at this time. Previous studies have shown [42] that individuals of this species are predominantly found in deeper waters after March, with larger body sizes, indicating a shift in fishing grounds toward deeper regions. The optimal 50 m st range is between 5.7 °C and 8.5 °C, with this parameter showing more variation from December to April but stabilizing in May and June. During these two months, 50 m st may be a crucial factor in the formation of central fishing grounds. The optimal SSH range is −14.5 to −7.1 cm, with sea surface height representing convergence and divergence of ocean currents. Changes in SSH can promote vertical mixing in the water column, bringing nutrient-rich waters to the surface and supporting primary productivity. The optimal SSS range for fishing grounds is between 33.8‰ and 34.0‰. SSS within fishing grounds almost consistently remains within this range, suggesting that suitable SSS is a necessary condition for squid fishing grounds. Salinity affects the osmotic balance of *Illex argentinus*, which in turn influences metabolism [43], indicating that this species has strict requirements for seawater salinity. The optimal MLD range includes several intervals: 13.1–19.2 m, 24.1–25.6 m, and 30.8–32.6 m. In studying Pacific fishing grounds, Gong [44] found that MLD significantly affects squid distribution. Given the proximity of the Southwest Atlantic high seas to Antarctica and the convergence of warm and cold currents, MLD fluctuates considerably, with depths ranging from under 10 m to over 100 m.

The CNN-Attention model’s predictions for 2024 also reflect the influence of key environmental factors on the squid fishing grounds. In December 2023, the distribution area of this squid was limited, with only general fishing grounds present. This is likely due to the low SST, below 9.1 °C, which is considerably outside the optimal SST range. As water temperatures rose, central fishing grounds began to form and gradually expanded to a larger scale. From January to February, SST was mainly between 10.8 °C and 14.2 °C, reaching the optimal SST range and sitting near the middle of this range, making SST highly favorable for the formation of central fishing grounds. In March, SST dropped below 10 °C again, and squid populations gradually moved southeast, expanding into deeper water areas. This movement aligns with their biological characteristics and seasonal migration patterns [45]. In the southeastern fishing grounds, 50 m water temperatures remained consistently between 6.1 °C and 8.5 °C after March, suggesting that squid resource abundance stayed high as 50 m st reached optimal levels. The SSS fluctuation range remained consistent with levels from 2020 to 2023, staying within a stable range. SSH was below −10 cm in December but increased in January and remained above −10 cm until the end of February. SSH then dropped again in March, reaching below −18 cm in April, falling outside the optimal SSH range. This trend in SSH shows some alignment with SST. In this region, MLD ranged from 12.1 m to 24.6 m before March 2024. After March, MLD increased gradually, reaching depths exceeding 60 m in certain areas by June. According to the model’s forecast, from December 2023 to February 2024, the fishing grounds were significantly influenced by SST, MLD, and SSH changes. During March to April 2024, 50 m st, MLD, and SSH were the primary influences, while after May, the species’ increased adaptability due to maturation allowed central fishing grounds to persist even as multiple environmental factors fell outside optimal ranges.

### 4.3. Comparison of Fishing Ground Prediction Effectiveness

Numerous studies have predicted squid fishing grounds across global oceans. For instance, Chen [46] and Cui [22] used a spatial resolution of 0.5° × 0.5° and a monthly temporal resolution to predict squid fishing grounds in the Northwest Pacific and Indian Oceans, achieving accuracies of 73.4% and 57.4%, respectively. However, the relatively coarse spatiotemporal scales used in these studies may limit prediction accuracy, and the use of fishing log data alone introduces significant time lags, limiting practical guidance for fishing operations. In contrast, this study utilized a finer resolution of 0.1° × 0.1° and monthly intervals, and vessel position data can be obtained in near real time, better meeting production needs.

The CNN-Attention model results show that the CNN-Attention model trained on vessel position data outperformed the model trained on fishing log data in fishing ground classification tasks, demonstrating the high reliability of vessel position data. Vessel position data are better suited for fishing ground classification than fishing log data, likely due to fundamental differences in the information each carries. As spatiotemporal data, vessel position data accurately reflect vessel activity patterns and the geographical distribution of fishing areas [47,48]. This information is directly valuable for distinguishing central from general fishing grounds, especially in terms of spatial locations and seasonal changes. Consequently, the model trained on vessel position data better captures differences among fishing grounds after extracting these spatiotemporal features, resulting in higher accuracy on the validation set. In contrast, although fishing log data contain information on catch amounts and fishing times, their spatial resolution and detail are limited compared to vessel position data. Fishing logs generally focus on recording catch results rather than the dynamic locations during fishing activities, which can make them less precise in describing fishing ground distributions. As a result, the model trained on fishing log data struggled to extract effective spatial features, leading to lower performance on the validation set, greater fluctuations in loss values, and lower model stability.

The fishing ground classification standard for the vessel position data model is based on trawl density, whereas the log data model uses CPUE. Given the training and validation set performance of both models, vessel position data benefit from advancements in remote sensing technology, providing more accurate temporal and spatial coordinates than fishing log data, which allows better matching with environmental information and leads to higher training accuracy and better generalization [49]. A comparison of each model’s 2024 predictions with actual outcomes showed that the fishing log model had high accuracy in December and June. In these months, unfavorable ocean conditions prevented the formation of large central fishing grounds, and the model’s learned behavior accurately predicted a high proportion of general fishing grounds. The fishing log model also maintained good accuracy in other months, suggesting that despite its lagging nature and some spatial inaccuracies, fishing log data can still reliably indicate fishing ground locations. The vessel position data model showed greater deviation from actual fishing grounds in the early and late stages of the fishing season. Before the squid populations reached a significant scale in the high seas in December, trawlers conducted trial fishing across various areas. In May and June, as the squid matured and became more adaptable to environmental changes, their distribution became more dispersed, prompting vessels to increase trawling density for economic purposes. Although using vessel position data to predict fishing ground levels based on trawl density introduced greater error at the start and end of the fishing season, its accuracy during peak season was high, even exceeding that of the log data model in certain months. This indicates that the approach proposed in this study achieves high accuracy during peak fishing seasons, and vessel position data can be effectively used to identify fishing grounds during this period or for single-species fishing grounds.

In summary, the advantage of fishing log data is their ability to accurately record catch amounts for each species, though some human-induced errors may exist. Vessel position data, in contrast, provide accurate spatial locations and can be obtained in near real time, eliminating the lag associated with fishing log data. However, when fish populations are dispersed, trawling density from vessel position data may not reliably represent fishing ground locations. This study suggests that integrating both data types in the future would combine their strengths. By using AIS vessel data to record vessel coordinates, and having fishermen promptly record catch species and quantities in a computerized system after each trawl, land-based units could use satellite communication to integrate both data types, effectively harnessing the advantages of each source.

## 5. Conclusions

This study used AIS vessel position data to determine the fishing ground distribution of this squid in the high seas of the Southwest Atlantic from 2020 to 2024. AIS data were combined with SST, SSH, and other ocean environment data to create datasets that were input into the CNN-Attention deep learning model for training and prediction validation. A comparison with fishing log-based results demonstrated that using vessel position data for fishing ground classification is scientifically sound and reliable during peak fishing season. Correlation analysis using the Spearman function further revealed interactions and trends among ocean environmental factors.

By constructing two CNN-Attention models, this study successfully predicted the dynamic changes in these squid fishing grounds for 2024. The prediction results show that, influenced by changes in ocean environmental factors like sea water temperature, the distribution of squid fishing grounds exhibits notable spatiotemporal variation throughout the fishing season. Model performance indicators (accuracy, precision, recall, and F1-score) confirmed that the CNN-Attention model has strong generalization ability and predictive effectiveness, providing high accuracy for predicting the squid habitats. The environmental data used in this study can be obtained in near real time with ease, and the near-real-time nature of vessel position data significantly reduces the lag associated with fishing log data in previous studies. This approach also proposes a new method for determining fishing ground levels, offering valuable support for addressing issues such as climate change adaptation, marine ecosystem protection, optimized fishing plans, and combatting illegal fishing. The method can also be applied to predict habitats and resource abundance for other marine species.

Despite advancements in fishing ground classification and analysis methods, this study has limitations. First, the vessel characteristics during net retrieval and deployment differ from those during fishing operations, so future work will apply more detailed thresholds to identify these specific times. Second, although *Illex argentinus* is the primary catch for trawlers in the high seas of the Southwest Atlantic from December to June, some trawls may yield only other species, such as hake. If future research incorporates video monitoring data to exclude trawls without the squid, this could further reduce classification error and enhance the model’s predictive accuracy for the squid fishing grounds.

## Data Availability

The marine environmental data used in this study can be downloaded from the following two websites: “http://apdrc.soest.hawaii.edu/las_ofes/v6/dataset?catitem=71 (accessed on 9 October 2024)” and “https://data.marine.copernicus.eu/products (accessed on 10 October 2024)”.
